# Magnetoelectric Force Microscopy on Antiferromagnetic 180^∘^ Domains in Cr_2_O_3_

**DOI:** 10.3390/ma10091051

**Published:** 2017-09-07

**Authors:** Peggy Schoenherr, L. Marcela Giraldo, Martin Lilienblum, Morgan Trassin, Dennis Meier, Manfred Fiebig

**Affiliations:** 1 Department of Materials, ETH Zurich, Vladimir-Prelog-Weg 4, 8093 Zürich, Switzerland; marcela.giraldo@mat.ethz.ch (L.M.G.); martin.lilienblum@mat.ethz.ch (M.L.); morgan.trassin@mat.ethz.ch (M.T.); manfred.fiebig@mat.ethz.ch (M.F.); 2 Department of Materials Science and Engineering, Norwegian University of Science and Technology, Sem Sælandsvei 12, 7034 Trondheim, Norway; dennis.meier@ntnu.no

**Keywords:** magnetoelectric force microscopy, antiferromagnetic domains, Cr_2_O_3_, second harmonic generation

## Abstract

Magnetoelectric force microscopy (MeFM) is characterized as methodical tool for the investigation of antiferromagnetic domain states, in particular of the 180${}^{\circ }$ variety. As reference compound for this investigation we use Cr${}_{2}$O${}_{3}$. Access to the antiferromagnetic order is provided by the linear magnetoelectric effect. We resolve the opposite antiferromagnetic 180${}^{\circ }$ domain states of Cr${}_{2}$O${}_{3}$ and estimate the sensitivity of the MeFM approach, its inherent advantages in comparison to alternative techniques and its general feasibility for probing antiferromagnetic order.

## 1. Introduction

Scanning probe microscopy (SPM) techniques have revolutionized nanoscience and nanotechnology. As local probe they can find links between the microstructure of a material and its electronic properties, as for example in the discovery of micro-patterns in 2D materials like graphene [1,2]. Other examples are found in life sciences where scientists have been able to visualize cell organelles and even manipulate single membrane proteins towards a better understanding of cellular interactions [3,4]. Not only the observation, but also the manipulation of matter at the nanoscale can be performed with SPM techniques, such as in the case of three-dimensional additive manufacturing [5].

Given the current technological advances and intriguing questions in nanotechnology, the demand for new characterization tools for analysis on the nanoscale increases continuously. In this sense, SPM techniques have undergone a tremendous and fast development in the last two decades [6]. The diversity of operational modes makes them versatile tools with a profound impact on materials science, physics, chemistry, life sciences and many other fields [6]. Their scope continues to expand and by now, SPM techniques undoubtedly constitute the most widely-used characterization tool on the nanoscale in research laboratories and industry.

SPM techniques proved particularly useful in the investigation of ferroic materials, allowing the observation of the ferroic state with a resolution on the order of ~20 nm where they revealed information that was hardly accessible before. Outstanding breakthroughs were made on almost all types of ferroics. This encompasses ferromagnetic nanostructures [7], ferroelectric domain structures [8,9] including the different electronic and transport properties at the domain walls [10,11] and also other varieties of ferroic order like ferroelasticity [12] or ferrotoroidicity [13].

Even though local ferroic properties have been widely studied by SPM, the approach to antiferromagnetic order, that is, magnetic long-range order with a compensated net magnetization, is almost non-existent. This is regrettable because antiferromagnets constitute the majority of magnetically ordered materials in nature [14,15] and they are connected to many fundamental states of matter involving strong electronic correlations such as superconductivity, colossal magnetoresistance, multiferroicity, quantum-critical behavior and others [16,17,18,19]. Even from the perspective of application, antiferromagnetic materials get increasing attention because of their robustness against external magnetic perturbations and their coupling effects to ferromagnetic states in the form of exchange bias or magnetoelectric heterostructures [14,20,21,22,23,24,25]. Since antiferromagnets do not exhibit a net magnetization, however, it is not trivial to access or control them. Efforts aimed at understanding the properties of antiferromagnets—their domain topography, interfacial coupling effects or nanoscale inhomogeneities—are thus very timely and maybe a key to their systematic manipulation.

In 2013, Geng and co-workers proposed a new SPM technique, termed “magnetoelectric force microscopy” (MeFM), that can provide access to certain antiferromagnetic structures [26,27,28]. The linear magnetoelectric effect (LME) denominates an electric field inducing a proportional magnetization, as well as a magnetic field inducing a proportional polarization [19,29]. The LME can occur in 58 out of the 90 magnetic point symmetry groups [29]. For example, it has been well studied on materials like orthorhombic GaFeO${}_{3}$ [30], tetragonal TbPO${}_{4}$ [31] or trigonal Cr${}_{2}$O${}_{3}$ [32]. In materials like these, the LME can be employed to convert antiferromagnetism into a SPM-measurable magnetization, just as piezoresponse force microscopy converts ferroelectricity into a SPM-measurable mechanical deformation. Geng et al. investigated hexagonal ErMnO${}_{3}$. For accessing the antiferromagnetic state of the compound, they applied a magnetic bias field that promoted a transition to an inhomogeneous and weakly ferromagnetic non-equilibrium state permitting the LME and allowing to perform MeFM on the domains at cryogenic temperature. This approach was extremely effective for spatially resolving the coexistence of ferroelectric and magnetic domains in hexagonal ErMnO${}_{3}$ and for gaining new insights into the local multiferroic properties of the hexagonal manganites. For establishing the *methodical* aspects of MeFM, however, a different approach is required. Such experiments would have to be performed on a well-known reference antiferromagnetic material, in the absence of external bias fields or interfering, coexisting forms of ferroic order. Preferably under ambient conditions and with simple domain structures. To provide such a methodical benchmark for MeFM is the purpose of our work.

Thus, in this paper we apply MeFM to Cr${}_{2}$O${}_{3}$, a reference antiferromagnetic material displaying the LME. We show that MeFM reveals the elusive type of 180${}^{\circ }$ antiferromagnetic domain patterns of Cr${}_{2}$O${}_{3}$ and we verify our results by direct comparison to non-linear optical experiments. Our experiments allow us to quantify the sensitivity of MeFM and to draw conclusions about its general feasibility and particular advantages. Along with this, we will discuss the transfer of our benchmark experiment to other materials and to the cryogenic range where antiferromagnetic ordering temperatures are often found. All this will further promote MeFM as a standard technique to investigate domain structures in the important class of antiferromagnetic materials for science as well as industry.

### Antiferromagnetic Cr${}_{2}$O${}_{3}$ and the Linear Magnetoelectric Effect

**Figure 1:**
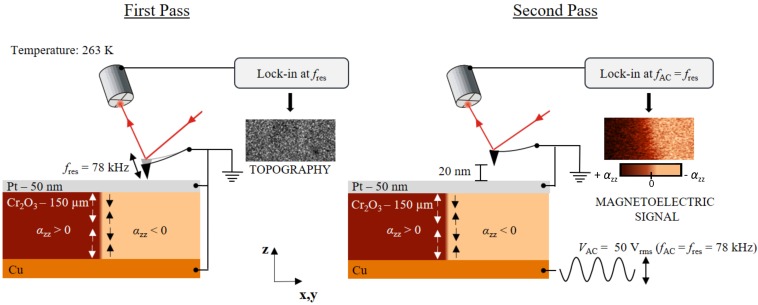
The principle of MeFM. In the first pass, the topography is detected in tapping mode with the tip oscillating at its resonance frequency ($f_{\mathrm{res}}$). To avoid electrostatic contributions, the whole system is grounded. In the second pass, the tip is lifted to a height of about 20 nm, and the mechanically driven oscillation is stopped. Instead, an AC voltage is applied to the back electrode. The applied voltage induces an oscillating magnetic field via the LME. Opposite 180${}^{\circ }$ domain states exhibit a relative phase difference in the magnetoelectric signal of 180${}^{\circ }$. This phase shift is detected by a lock-in amplifier and transferred into a contrast between different antiferromagnetic domains in the second pass.

A variety of techniques for the visualization of antiferromagnetic domains is already known [33], but these are usually based on secondary effects like magnetostriction and thus not feasible for the distinction of 180${}^{\circ }$ antiferromagnetic domain states, which differ by a mere reversal of all the magnetic moments in the unit cell (see Figure 1). Up to now, there are only very few techniques which are able to distinguish 180${}^{\circ }$ domains, like polarized neutron topography [34,35], optical second harmonic generation (SHG) [36,37], certain linear magnetooptical effects [38,39] or X-rays diffraction imaging [40]. A drawback of these techniques is that they are small in signal, expensive or limited in resolution.

By using the LME, such restrictions may be overcome, and even the elusive antiferromagnetic 180${}^{\circ }$ domains would be accessible to SPM. The LME denotes a direct coupling F∝αEH between electric (E) and magnetic (H) fields in matter with α as the magnetoelectric coupling coefficient and *F* as the resulting free-energy contribution [41,42]. Derivation of *F* leads to linear cross-coupling between the magnetic field and the induced polarization (P∝αH) and between the electric field and the induced magnetization (M∝αE). An intuitive model to imagine the latter relation for antiferromagnets is that the electric field displaces up- and down-spin atoms in an originally identical environment such that the environment for up- and down-spin ions becomes different. As a result, the up- and down-magnetizations no longer cancel and a small net magnetization emerges in an electric field [43].

For a methodical benchmark of the distinction of antiferromagnetic domains, especially of the 180${}^{\circ }$ type, by MeFM via the LME, we ideally require a material in which the antiferromagnetic order is directly relating to the LME and not obscured by other, coexisting forms of long-range order. Cr${}_{2}$O${}_{3}$ is a prototypical material exhibiting a LME with 180${}^{\circ }$ antiferromagnetism as its only type of origin. Both the antiferromagnetic order and the resulting LME of Cr${}_{2}$O${}_{3}$ are well understood [32,44]; in fact, the LME was first theoretically predicted and experimentally observed for this material [45,46,47]. Cr${}_{2}$O${}_{3}$ is trigonal and consists of Cr${}^{3+}$ ions in a distorted octahedral O${}^{2-}$ cage. As sketched in Figure 1, the magnetic Cr${}^{3+}$ moments of the unit cell form an antiferromagnetic ↑↓·↑↓ (domain state ‘+’) or ↓↑·↓↑ (domain state ‘−’) easy-axis arrangement below the Néel temperature (307.6 K) that breaks time-reversal symmetry and permits the LME (the dot denotes an empty O${}^{2-}$ octahedron).

The LME of Cr${}_{2}$O${}_{3}$ exhibits a non-linear temperature dependence, displaying the largest magnetoelectric coefficient, $\alpha_{zz}=4.31$ ps/m, along the trigonal *z*-direction at about 263 K [48]. Note that much higher magnetoelectric coupling coefficients are observed in composite systems and multiferroics [42,49], one of the reasons why such materials have been experiencing a drastic increase of attention over the past two decades [42]. These magnetoelectric coupling coefficients, however, are fundamentally different from the original LME that is the basis of our MeFM technique.

In summary, Cr${}_{2}$O${}_{3}$ constitutes the optimal benchmark material to verify MeFM as a novel SPM technique that even provides access to the elusive type of 180${}^{\circ }$ antiferromagnetic domains.

## 2. Research Method and Materials

### 2.1. Cr${}_{2}$O${}_{3}$ Samples

Cr${}_{2}$O${}_{3}$ bulk samples are grown by the Verneuil method and oriented by Laue diffraction. The samples are cut perpendicular to the *z*-axis (i.e., (001) orientation) using a diamond saw. Afterwards, the samples are thinned down to 150 μm by lapping with SiC powder and chemo-mechanically polished with a colloidal silica slurry to reveal surfaces with a root-mean-square roughness of about 3 nm. For the application of an electric field between the two sides of the sample for the SPM measurements, the sample is fixed on a copper plate and covered on the opposite side with a Pt layer of 50 nm. The deposition is done at room temperature using DC magnetron sputtering at 10 W and argon as sputtering gas at $5\times 10^{-2}$ mbar. To assure adhesion between the Pt layer and the Cr${}_{2}$O${}_{3}$ surface, a Ti layer of 2 nm is deposited in beforehand by sputtering at 20 W under the same atmospheric conditions as for platinum. We routinely verify the LME of our Cr${}_{2}$O${}_{3}$ by magnetoelectric current measurements before beginning with the SPM experiments.

### 2.2. Magnetoelectric Force Microscopy

We apply a two-pass line-by-line MeFM technique using a magnetic cantilever. Figure 1 shows the operation mode of the technique. The first pass records the topography in tapping mode, where the cantilever is excited mechanically to oscillate at its resonance frequency ($f_{\mathrm{res}}=78$ kHz). During the first pass, all metallic layers, as well as the tip, are grounded in order to suppress electrostatic contributions.

In the second pass, the tip is lifted up by 20 nm, retracing the topography measured in the first pass, while the forced mechanical oscillation is turned off. Note that the lift height is not corrected by the oscillation amplitude of the tapping mode since the latter is not known. For inducing the magnetoelectric response, a bias AC voltage with a maximum of 50 V${}_{\mathrm{rms}}$ is applied to the copper back electrode whereas the Pt top electrode and tip remain grounded. The electric field within the Cr${}_{2}$O${}_{3}$ sample stimulates a magnetic response via the LME. The electric-field-induced magnetization is sensed by a magnetically coated tip; electrostatic contributions are suppressed by the grounded metallic top layer. Sensitivity is greatly increased by choosing the frequency $f_{\mathrm{AC}}$ of the bias voltage to match the mechanical resonance $f_{\mathrm{res}}$ of the cantilever. The sign of the coefficient α parameterizing the LME depends on the antiferromagnetic $180^{\circ }$ domain state of the sample. Moving between opposite domain states reverses the sign of α which leads to a $180^{\circ }$ phase shift in the induced AC magnetization. In contrast, the amplitude of α is determined by the strength of the electrically induced magnetic field. The induced tip oscillation is detected by a lock-in amplifier in Cartesian coordinates. The lock-in amplifier signal is adjusted so that amplitude and phase information are both shown in the X-channel. The sign of the signal from the X-channel represents the sign of the magnetoelectric constant α, and is encoded as brightness as indicated by the scale bar in Figure 1.

MeFM measurements are carried out using a commercial NT-MDT device with a home-build cooling system consisting of a water-cooled three-stage Peltier element [50]. The experiments are performed in a nitrogen gas environment at 263 K, where the magnetoelectric coefficient $\alpha_{zz}$ of Cr${}_{2}$O${}_{3}$ reaches its maximum value [47]. Standard magnetic tips (PPP-MFMR, Nanosensors, Q-factor 100–200) with an apex diameter below 50 nm are used and pre-magnetized to enhance the sensitivity.

In the MeFM images shown in the following sections, we added up forward and backward traces and apply standard image processing to enhance the image quality. For the quantitative evaluations, we only use the raw data of the forward traces with a zero-order line fit.

### 2.3. Second Harmonic Generation

Laser-optical SHG, that is, frequency doubling of a light wave in a material, is used to spatially resolve the antiferromagnetic domain structure of the Cr${}_{2}$O${}_{3}$ bulk samples. At the same time, it allows us to compare the results from the SHG and the MeFM approach and highlight the particular benefits of the latter. SHG in the leading order becomes allowed when inversion symmetry is broken [51]. It is therefore the ideal tool to detect ferroic structures [52], including in particular 180${}^{\circ }$ domain states, if the ferroic order breaks inversion symmetry. For antiferromagnetic systems this was demonstrated for the first time in Cr${}_{2}$O${}_{3}$ with its non-centrosymmetric ↑↓·↑↓ spin order [36,37]. The SHG images are convenient for identifying the position of the antiferromagnetic 180${}^{\circ }$ domains as the domain size in Cr${}_{2}$O${}_{3}$ is in the order of hundreds of micrometers.

To acquire the SHG images, bulk Cr${}_{2}$O${}_{3}$ samples are excited in transmission and at normal incidence by an unfocused circularly polarized laser beam with a photon energy of 1.033 eV and a pulse energy of 30 μJ. A camera lens is used to collect the SHG signal. Optical filters are added to select the spectral region of interest and to suppress scatter laser light generated in the optical components and higher-harmonic contributions. We use a Coherent Elite Duo laser system with optical parametric amplifier, which emits 120 fs pulses at a repetition rate of 1 kHz. SHG light is detected at room temperature with a Jobin Yvon Back Illuminated Deep Depletion digital camera with a near-infrared detector chip of 1024×256 pixels with 100% filling factor. To reduce noise, the camera is cooled with liquid nitrogen [36].

Topographic features on the sample surface that are visible with conventional light microscopy and by SHG help us to localize the same area on the sample for the SHG and MeFM measurements and we can thus compare the results on the antiferromagnetic domain structure obtained with the two techniques.

## 3. Results and Discussion

### 3.1. Detection of Antiferromagnetic 180${}^{\circ }$ Domains

**Figure 2:**
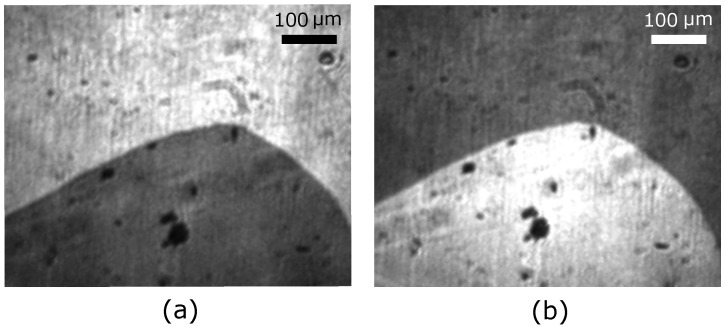
SHG images at room temperature of a *z*-oriented Cr${}_{2}$O${}_{3}$ bulk crystal prior to the deposition of the Pt electrode. (**a**) Exposure with circularly left-handed fundamental light; (**b**) Exposure with circularly right-handed fundamental light. Two antiferromagnetic domain states are clearly distinguishable and exhibit the expected reversal of contrast with reversal of the circular polarization [37].

SHG images prior to the deposition of the Pt electrode are depicted in Figure 2. Dark and bright areas in the images correspond to opposite antiferromagnetic 180${}^{\circ }$ domain states of a *z*-oriented Cr${}_{2}$O${}_{3}$ sample. The contrast between the domains is obtained by the interference of the SHG wave induced by the antiferromagnetic order with the SHG wave emitted by the crystal lattice [36,52]. By changing the circular polarization of the incident laser light, the brightness of the two domain states is reversed, as expected [36,52].

**Figure 3:**
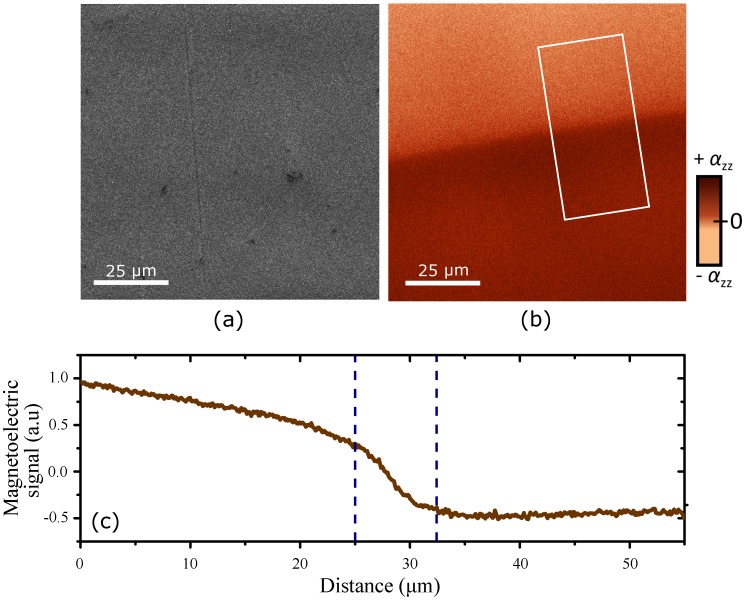
MeFM scans at 263 K on an area of 100×100 μm${}^{2}$ of a *z*-oriented Cr${}_{2}$O${}_{3}$ sample as in Figure 2. (**a**) First pass—topography; (**b**) Second pass—magnetoelectric signal. The antiferromagnetic domain wall is only visible in the magnetoelectric scan; (**c**) Cross-section of the region at the domain wall as outlined in panel (**b**). The markers in (**c**) indicate a width of the wall region of about 7.5 μm which is a convolution effect caused by the tilted propagation of the wall away from the surface (see Section 3.5).

Figure 3a,b display 100 × 100 μm${}^{2}$ MeFM scans of the topography (first pass) and of the electrically induced magnetic signal (second pass), respectively, in the vicinity of an antiferromagnetic domain wall whose location has been determined by topographic features as described above. The scanned area is recorded with a pixel size of ~100 nm and an integration time per point of 20 ms. Figure 3a reveals the topography in a flat area of the sample. Surface roughness in this area has a root-mean-square value of about 3 nm and does not exhibit any change across the domain wall, as expected for 180${}^{\circ }$ domain states. The topography of the surface is an input for the second pass in which the tip retraces the topography 20 nm away from the sample. By doing this, the tip is exposed to a constant background force, mostly from Van-der-Waals interactions, as well as more sensitive to long-range forces, such as the magnetization induced via the LME in the present case.

The image in Figure 3b shows the signal of the Cartesian X-channel from the lock-in amplifier in the second pass of the MeFM technique. In contrast to the topography scan, we observe a striking discontinuity at the position of the domain wall. Two regions with different brightness are clearly distinguishable with a gradual change of contrast around the position of the domain wall on a length scale of ~7.5 μm according to the cross-section in Figure 3c (this large width and the slope in the domain brightness will be discussed below). This contrast is explained as follows. The AC bias voltage applied to the sample induces an oscillating electric field along the *z*-direction of the Cr${}_{2}$O${}_{3}$ crystal. This is converted into an AC magnetic field by the LME. At the domain wall, the magnetic field experiences a 180${}^{\circ }$ phase shift due to sign reversal of the magnetoelectric constant α between the opposite antiferromagnetic domain states. The measured signal in Figure 3b confirms that MeFM distinguishes very clearly between the antiferromagnetic 180${}^{\circ }$ domain states of our reference compound Cr${}_{2}$O${}_{3}$.

### 3.2. Verification of the Magnetoelectric Detection

**Figure 4:**
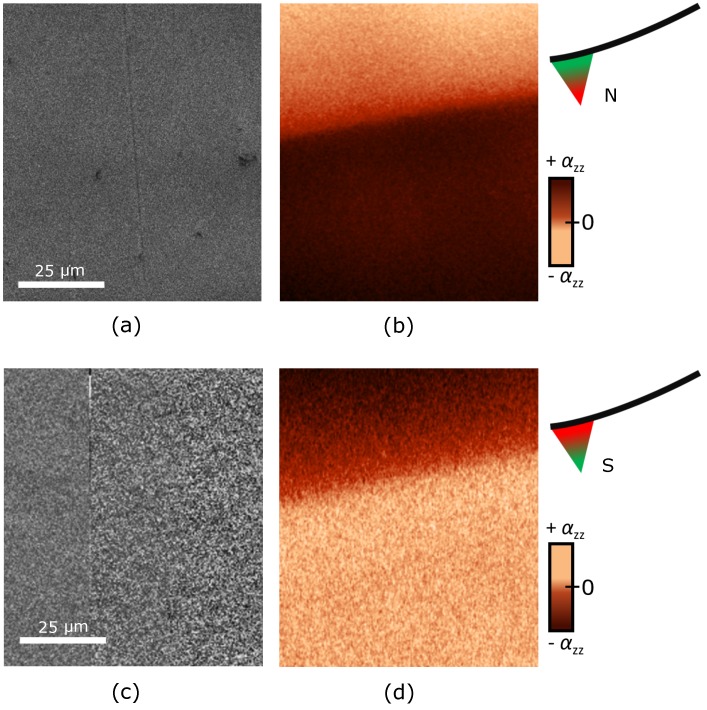
Effect of tip magnetization reversal. Pairs of topography and magnetoelectric images at 263 K as in Figure 3 with (**a**,**b**) original tip magnetization (100 nm image resolution) and (**c**,**d**) reversed magnetization (400 nm image resolution). Identical areas were scanned before and after tip remagnetization; the relocation procedure for the tip is described in the text.

Albeit Figure 3b reveals a stark change of brightness between opposite antiferromagnetic domain states, it does not show to what extent other effects interfere with the LME response. In order to scrutinize this issue, we switch the tip magnetization. For this, the tip is removed from the SPM system and its magnetization is saturated using a permanent magnet. Afterwards, the tip is re-mounted to scan the probed area a second time. Relocation of this area is accomplished by a combination of optical-microscopy and topography scans, both available with our SPM system, beyond the limited area show in Figure 4. The tip magnetization reversal has to reverse the contrast in the LME response whereas any residual topographic or electrostatic forces between the tip and the electrodes on the sample would be observable as tip-magnetization-independent contributions.

Figure 4a,c depict the topography scans with the corresponding magnetoelectric scans at opposite tip magnetization displayed in Figure 4b,d. We show both topographic images to confirm that in neither any feature of the antiferromagnetic domain boundary appears. In contrast, a reversal of brightness with reversal of the tip magnetization is observed in the magnetoelectric scans. We therefore conclude that the signal in Figure 4b,d is only induced by the LME and hence of purely antiferromagnetic origin.

### 3.3. Sensitivity

Now that we know that the magnetoelectric signal in Figure 3 and Figure 4 is entirely related to the antiferromagnetic order, let us have a look at the sensitivity of the MeFM scan as parameterized by the signal-to-noise ratio (SNR). Typical sources of noise result from random fluctuations generated by the electronic devices in the detection system (photodiode), from mechanical readout instabilities of the lock-in amplifier, or from thermal noise-driven fluctuations of the cantilever. Other factors may be inhomogeneities at the Cr${}_{2}$O${}_{3}$-Pt interface or in the Pt layer itself, as well as instabilities in the cantilever-tip-surface system.

**Figure 5:**
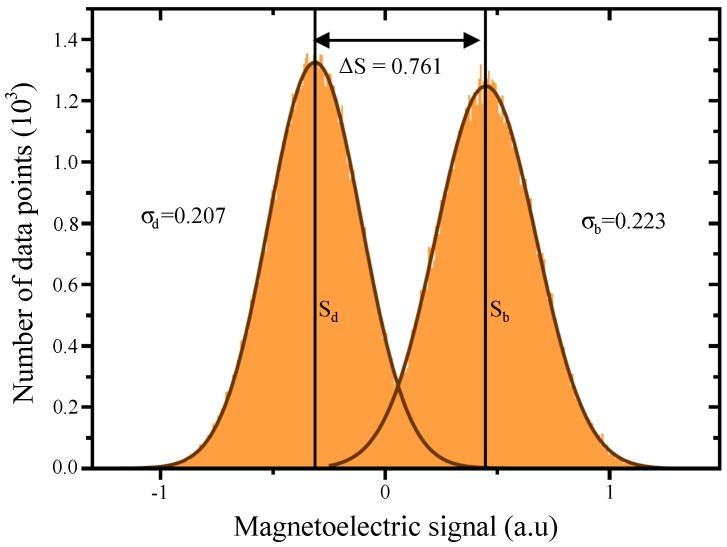
Histogram taken in an area of 900 μm${}^{2}$ in the two opposite antiferromagnetic domain states of the magnetoelectric scan in Figure 3b. Their average values $S_{b,d}$ correspond to the magnetoelectric signal of the bright and the dark domain, respectively, and the corresponding standard deviations $\sigma_{b,d}$ parametrize the noise of the measurement.

To determine the SNR, Figure 5 shows a histogram of MeFM data points taken on a homogeneous area of 900 μm${}^{2}$ in both antiferromagnetic domains of Figure 3b. Both histograms are fitted by a Gaussian function. Their average values are $S_{b}$ and $S_{d}$ with $\Delta S=S_{b}-S_{d}$; and the respective standard deviations are $\sigma_{b}$ and $\sigma_{d}$ with σ as their average. We find ΔS=0.761 and σ=0.215 which reveals a SNR of 3.5. In turn, this means that with the setup and scanning parameters applied for Figure 3b, we can detect a magnetoelectric coefficient down to α=1.2 ps/m. In comparison to other magnetoelectric antiferromagnets of recent interest, the LME in Cr${}_{2}$O${}_{3}$ is relatively weak (GaFeO${}_{3}$: $\alpha_{zz}=13.8$ ps/m, LiCoPO${}_{4}$: $\alpha_{yx}=30.6$ ps/m, TbPO${}_{4}$: $\alpha_{xy}$ = 730 ps/m [30,31,49,53]). We can therefore conclude that the sensitivity of our MeFM setup is well in the range that allows convenient detection of antiferromagnetic domains in materials exhibiting typical values of α.

In many materials, the magnetic ordering temperature, where the LME appears, may lie below room temperature. Measurements with non-cryogenic SPM equipment can be performed down to at least $-80{\;}^{\circ }$C [54] with a Peltier-cooled sample stage. But even the transfer of MeFM to cryogenic SPM systems is feasible, albeit more elaborate.

### 3.4. Possible Improvements

There are several ways to improve the sensitivity of the MeFM setup further. In the following, we will mention the most important ones. (i) Increasing the electric field will result in a larger induced magnetic field and, thus a larger MeFM response. This can be achieved by increasing the applied voltage or decreasing the sample thickness. The limiting factor is the dielectric constant of the material as it defines the break-down voltage; (ii) The LME typically shows a pronounced temperature dependence [48] so that the magnetoelectric signal can be optimized by choosing the temperature where α reaches its maximum value; (iii) Another important component are the electrodes, whereby the top electrode is more crucial. For our experiments we used a Pt top layer of 50 nm for applying the electric field and to shield the tip from electrostatic forces. By testing different thickness values and different electrode materials the interface quality, the shielding properties and the magnetic field permeability could be further improved; (iv) Tips with higher magnetic moments may be used which would lead to a stronger force between the tip and the magnetoelectrically induced magnetic field. Standard SPM systems can detect forces down to 1 pN [55,56]; (v) All the improvements so far enhance the signal strength. On the other side, the noise can be reduced by increasing the averaging time per data point or by measuring the same area several times. Note that in our work we already added forward and backward traces. If the antiferromagnetic domains formed by a sample have a lateral extension far above the resolution of the MeFM technique (as, e.g., in Cr${}_{2}$O${}_{3}$) the SNR may be improved by applying smoothing filters to the data. This reduces the statistical noise on the cost of (then expendable) spatial resolution. In summary, by choosing a sample with a thickness of 50 μm and *V* = 500 V${}_{\mathrm{rms}}$ (E≈15 kV/cm), which are accessible values for SPM [26], we conservatively estimate that we will eventually be able to detect a magnetoelectric constant down to 0.01–0.1 ps/m.

### 3.5. Superior Aspects of Magnetoelectric Force Microscopy

The direct comparison of MeFM and SHG in probing antiferromagnetic 180${}^{\circ }$ domains not only allows us to quantify the sensitivity of the MeFM approach. Moreover, it reveals a variety of aspects where MeFM is superior to the nonlinear optical characterization.

First of all, since our model compound Cr${}_{2}$O${}_{3}$ tends to form domains with an expansion of several 100 μm, we did not discuss the aspect of spatial resolution so far. MeFM with conventional tips will be capable of resolving antiferromagnetic domain structures with a lateral spatial resolution of 30 nm. With specially formed tips the resolution can be pushed further down to only 10 nm [57,58], which exceeds the resolution limit of optical experiments by two orders of magnitude.

Second, let us return to Figure 3b, where we observed that the brightness across the antiferromagnetic domain wall does not change as a step between two constant levels, but as a gradual transition across 7.5 μm with additional modulations of the magnetoelectric amplitude further away from the wall. We can exclude domain relaxation as a reason for this because the domain structures resolved by MeFM and SHG coincide and are thus stable. Furthermore, both the typical width of a magnetic domain wall and the resolution of the SPM experiment are far below 7.5 μm. Most likely, the gradient results from a depth effect since the induced magnetic field emerges from the whole sample. We suspect that the domain wall is slightly tilted with respect to the surface normal which would exactly lead to a brightness gradient as the one we observe. For a sample with a thickness of 150 μm we thus derive an inclination angle of the domain wall of about ~3${}^{\circ }$, under the additional assumption that the wall is going all the way straight through the sample.

**Figure 6:**
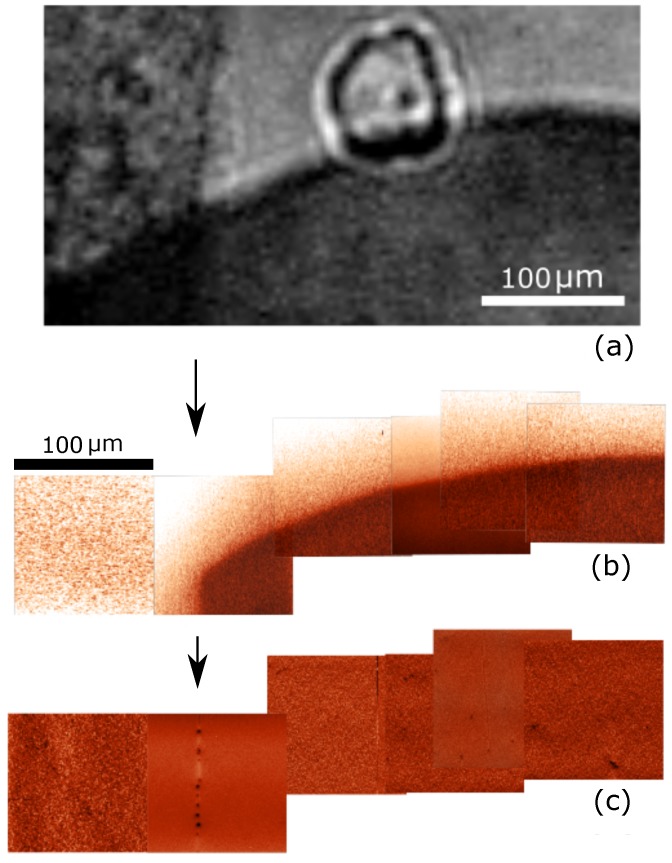
Images of the same region of a *z*-oriented Cr${}_{2}$O${}_{3}$ sample showing the spatial distribution of (**a**) SHG; (**b**) magnetoelectric signal; (**c**) topography. The arrows mark a scratch on the sample surface that extends into a crack within the bulk of the sample (The circular shaped object in the SHG image is a dust particle which was removed prior to the deposition of the Pt top electrode).

Finally, let us compare the MeFM and the SHG approach for a non-ideal Cr${}_{2}$O${}_{3}$ sample, with crystallographic defects. Images taken by either technique in the vicinity of an antiferromagnetic domain wall are shown in Figure 6a,b. Towards the right-hand side, the SHG image clearly exhibits two domain states. Towards the left-hand side, however, the domain contrast dissolves into a dark gray area for which unique association to an antiferromagnetic domains is not possible anymore. We therefore measured the same sample area by MeFM. Figure 6b is assembled from seven individual scans of 100 × 100 μm${}^{2}$. Note that they reproduce the position of the domain wall on the right, but in contrast to the SHG measurements two domain states are also clearly visible on the left.

In order to clarify this controversy, we compare the topography scans of the MeFM measurements (Figure 6c) with the magnetoelectric signal and the SHG images. We see that the topography reveals a surface defect on the sample which is marked by an arrow. This defect indicates a crack extending into the bulk of the sample. The crack scatters the fundamental laser light so that the corresponding region in the SHG image is shadowed and does not display the correct antiferromagnetic domain distribution. MeFM, on the other hand, does not suffer from this scattering effect because the electrically induced magnetic fields are not suffering from the shadowing effect as the transmitted light and are therefore less sensitive to crystal inhomogeneities.

## 4. Conclusions

In summary, we have presented a methodical characterization of MeFM as a probe for the spatially resolved detection of antiferromagnetic 180${}^{\circ }$ domains taking advantage of the LME, using Cr${}_{2}$O${}_{3}$ as our reference compound. Comparison to the characterization of the antiferromagnetic 180${}^{\circ }$ domains by SHG allowed us to identify intrinsic advantages of the MeFM approach in comparison to the nonlinear optical technique. Antiferromagnetic domain states in Cr${}_{2}$O${}_{3}$ are resolved with a SNR of 3.5. This allows to distinguish antiferromagnetic domain states in compounds exhibiting a magnetoelectric coefficient of at least 1 ps/m, but we discussed that further improvements of the MeFM approach may eventually allow us to reach a sensitivity of 0.01 to 0.1 ps/m. This covers all the known bulk compounds displaying a LME and may even be sufficient to resolve antiferromagnetic domain structures in complex new material classes like type-II multiferroics or magnetoelectric heterostructures.
